# An Extended Affinity Propagation Clustering Method Based on Different Data Density Types

**DOI:** 10.1155/2015/828057

**Published:** 2015-01-21

**Authors:** XiuLi Zhao, WeiXiang Xu

**Affiliations:** ^1^State Key Laboratory of Rail Traffic Control and Safety, Beijing 100044, China; ^2^Business School, Qilu University of Technology, Jinan 250353, China; ^3^School of Traffic and Transportation, Beijing Jiaotong University, Beijing 100044, China

## Abstract

Affinity propagation (AP) algorithm, as a novel clustering method, does not require the users to specify the initial cluster centers in advance, which regards all data points as potential exemplars (cluster centers) equally and groups the clusters totally by the similar degree among the data points. But in many cases there exist some different intensive areas within the same data set, which means that the data set does not distribute homogeneously. In such situation the AP algorithm cannot group the data points into ideal clusters. In this paper, we proposed an extended AP clustering algorithm to deal with such a problem. There are two steps in our method: firstly the data set is partitioned into several data density types according to the nearest distances of each data point; and then the AP clustering method is, respectively, used to group the data points into clusters in each data density type. Two experiments are carried out to evaluate the performance of our algorithm: one utilizes an artificial data set and the other uses a real seismic data set. The experiment results show that groups are obtained more accurately by our algorithm than OPTICS and AP clustering algorithm itself.

## 1. Introduction

Affinity propagation (AP) is a new partitioning clustering algorithm proposed by Frey and Dueck in 2007 [1]. By being different from the traditional partitioning clustering methods, AP clustering algorithm does not need to specify the initial cluster centers but automatically finds a subset of exemplar points which can best describe the data groups by exchanging messages. The messages to be exchanged among the data points are based on a set of similarities between the pairs of data points. AP algorithm assigns each data point to its nearest exemplar, which results in a partitioning of the whole data set into some clusters. AP algorithm converges in the maximization of the overall sum of the similarities between data points and their exemplars.

Most clustering algorithms store and refine a fixed number of potential cluster centers, while affinity propagation does not need them. AP algorithm equally regards all data points as potential exemplars (also named cluster centers). Among the data points there are two kinds of messages to be exchanged: the availability and the responsibility. The former one sent from point *k* to point *i* indicates how much support point *k* has received from other points for being an exemplar. The latter one sent from point *i* to point *k* indicates how well-suited point *k* is as an exemplar for point *i* in contrast to other potential exemplars. When the sum of the responsibilities and availabilities is maximized, the proceeding of the message-passing ends and a clear set of exemplars and clusters emerge.

**Figure 1:**
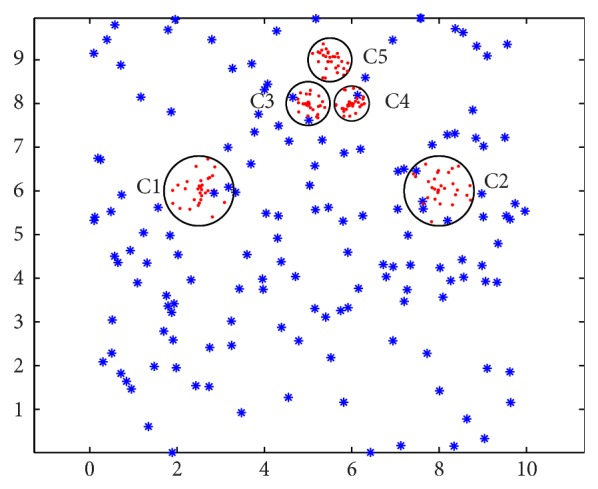
Data set.

**Figure 2:**
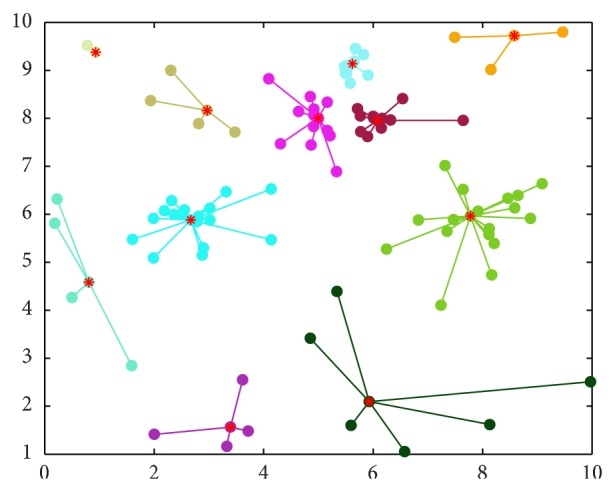
The clusters by AP.

**Figure 3:**
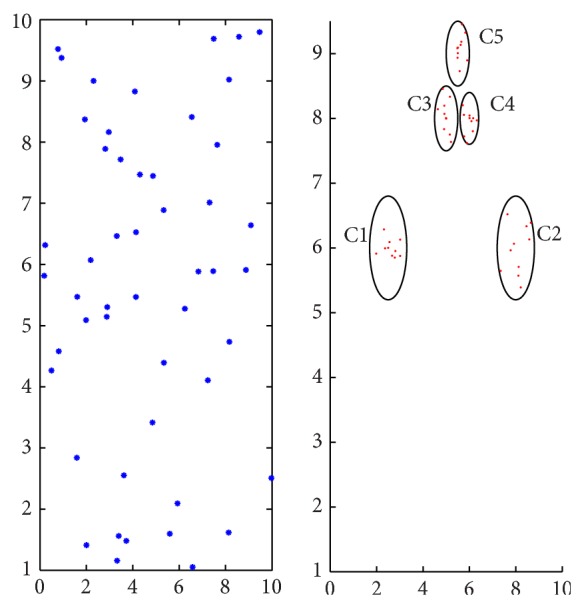
The ideal clusters.

But the AP clustering algorithm cannot deal with the nested clusters, which have different data density types. The toy example in Figure 1 illustrates clusters C1, C2, C3, C4, and C5 with more intensive data, but AP clustering algorithm cannot recognize them.  Figure 2 shows the result of AP clustering algorithm, which is in disagreement with the reality. Obviously, we want to obtain the clusters as shown in Figure 3. In this paper we propose an extended AP clustering algorithm that can cluster data point set into clusters according to their different density types. There are two novelties in our new extended AP clustering algorithm: (1) according to the frequency distribution curve of the nearest neighbor distance, it can identify the number of the different density types in the whole data set; (2) it can partition the data set into clusters more effectively than the OPTICS and AP clustering algorithm itself.

**Figure 4:**
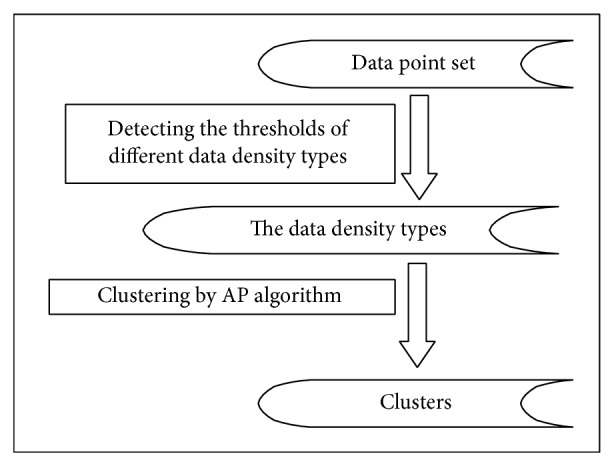
The two-step process for discovering clusters with different densities.

For the sake of simplicity, we design a simple toy data set to explain the context of our method, as shown in Figure 1. In this data set, there are some unknown data density types, and each of them may be nested to other clusters with other density types. Firstly, we transform data point set into a distance matrix and recognize the point density types according to the nearest neighbor distance matrix. Then we use AP clustering algorithm to classify the data points into several clusters. The whole clustering process consists of two steps (as shown in Figure 4). The first step is to identify dividing value of data density, and the second step is to establish the mechanism to form clusters.

This paper is organized as follows: in Section 2 some related works on AP clustering algorithm and some concepts are briefly introduced; in Section 3 the new semisupervised AP clustering algorithm is illustrated in detail. In Section 4 the proposed algorithm is applied to cluster some data sets both simulated and real data, and, finally, some conclusions are summarized in Section 5.

## 2. Related Work

### 2.1. Related Work on AP Cluster Methods

The goal of cluster analysis is to group similar data into some meaningful subclasses (or named clusters). There are three main categories in the clustering methods: partitioning, hierarchical, and density-based.

The first clustering category tries to obtain a rational partition of the data set that the data in the same cluster are more similar to each other than to the data in other clusters. Usually the partitioning criterion takes the minimum sum of the Euclidian distance.* K*-means and* K*-medoids are the classical partitioning cluster methods.

The second clustering category works by grouping data objects into a dendrogram tree of cluster. This category can be further classified into either merging (bottom-up) or splitting (top-down). The former one begins by placing each data point in its own cluster and then merges these atomic clusters into larger and larger clusters until certain termination conditions are satisfied. The latter one does reverse to the merging one by starting with all data points as a whole cluster and then subdivides this cluster into smaller and smaller pieces until it satisfies certain termination conditions (Han et al. 2001) [2].

Differing from the above clustering methods, the density-based clustering algorithms can discover intensive subclasses based on the similar degree between a data point and its neighbors. Such subclass constitutes more dense local area [3, 4].

In recent years, as a new clustering technique, AP clustering algorithm, proposed by Frey and Dueck in 2007, mainly depends on message passing to obtain clusters effectively. During the clustering process, AP algorithm firstly considers all data points as potential exemplars and recursively transmits real-valued messages along the edges of the network until a set of good centers are generated.

In order to speed up convergence rate, some improved AP algorithms have been proposed. Liu and Fu used the constriction factor to regulate damping factor dynamically and sped up the AP algorithm [5]. Gu et al. utilize local distribution to add constraint like semisupervised clustering to construct sparse similarity matrix [6]. Wang et al. propose an adaptive AP method to overcome inherent imperfections of the algorithm [7]. In the recent years AP algorithm has been applied in many domains, and it effectively solves many practical problems. These domains are included in image retrieval [8], biomedicine [9], text mining [10], image categorization [11], facility location [12], image segmentation [13], and key frame extraction of video [14].

### 2.2. Related Work on the Nearest Neighbor Cluster Method

#### 2.2.1. The Nearest Neighbor Distance

Let *X* = {*x*_1_ ⋯ *x*_*n*_} be a data set containing *n* data point objects and let {*C*_1_,…, *C*_*k*_, *k* ≪ *n*} be the types of different density. For simplicity and without any loss of generality, we consider just one of the density types* C*_1_.

Suppose that all data points in the* C*_1_ are denoted as {*p*_*i*_ : *p*_*i*_ ∈ *C*_1_, *i* = 1,…, *m*}. The nearest distance (named* D*_*m*_) of* p*_*i*_ is defined as the distance between* p*_*i*_ and its nearest neighbor. All* D*_*m*_ forms a distance matrix* D*. Because* C*_1_ obeys a homogenous Poisson distribution, the nearest distance matrix* D*_*m*_ also has an equivalent likelihood distribution with constant intensity (*λ*). For a randomly chosen data point* p*_*i*_ its nearest distance array* D*_*m*_ has a cumulative distribution function (cdf) as shown in the following formula:

(1) $$\begin{array}{c} \;\;G_{D_{m}}\left(d\right)=P\left(D_{m}\geq d\right)=1-\overset{m-1}{\underset{l=0}{\sum }}\frac{e^{-\lambda \pi d^{2}}\left(\lambda \pi d^{2}\right)}{l!}. \end{array}$$

The formula is gained by supposing a circle of radius *d* centered at a data point. More detailed, if* D*_*m*_ is longer than* d*, there must be one of 0, 1,2,…, *m* − 1 data points in the circle. Thus the* pdf* of the nearest distance *D*_*m*_ (*d*; *m*, *λ*) is the derivative of *G*_*D*_*m*__ (*d*), as shown in the following formula:

(2) $$\begin{array}{c} f_{Xm}\left(x;m,\lambda \right)=\frac{e^{-\lambda \pi x^{2}}2{\left(\lambda \pi \right)}^{m}x^{2m-1}}{\left(m-1\right)!}. \end{array}$$

#### 2.2.2. Probability Mixture Distribution of the Nearest Distance

Hereafter, we will discuss the situation of multiple data density types with various intensive data overlap in a local area. A mixture distribution can be modeled for such data points with different density types. As shown in Figure 1, there are two density types in the toy experiment: three clusters in one data density type, and two clusters in another data density type, as well as plenty of background noise data. According to the nearest distance matrix* D*, a histogram can be drawn to show the different distribution of the different density types. In order to eliminate the disadvantage of the edge effect, we transform the data point into toroidal edge-corrected data; thus the points near the edges will have the same mean value of the nearest distance with the data points in the inner area. A mixture density can be assumed to express the distribution for *k* types of data point density; the equation is shown as follows:

(3) $$\begin{array}{c} D_{m}\sim \overset{k}{\underset{i=1}{\sum }}w_{i}f\left(d;m,\lambda_{i}\right)=\overset{k}{\underset{i=1}{\sum }}w_{i}\frac{e^{-\lambda_{i}\pi x^{2}}2{\left(\lambda_{i}\pi \right)}^{m}d^{2m-1}}{\left(m-1\right)!}. \end{array}$$

The weight of the *i*th density type is represented by the symbol *w*_*i*_ (*w*_*i*_ > 0 and ∑*w*_*i*_ = 1). The symbol *m* means the distance order and *λ*_*i*_ is the *i*th intensity type. To a given data point *p*_*i*_ there is a one-to-one counterpoint of its nearest distance* D*_*m*_, and data points can be grouped by splitting the mixture distribution curve; in other words, the number (*k*) of density types and their parameters (*w*_*i*_, *λ*_*i*_) can be determined.

**Figure 5:**
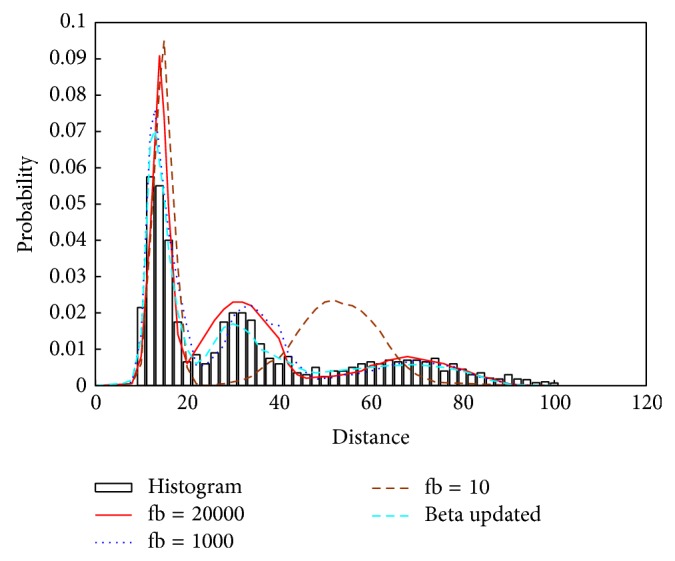
Histogram of the nearest distances.

Figure 5 shows the histogram of the nearest distance of the toy data set and the fitted mixture probability density function, where fb is defined as the expectation of the parameter *λ*_*i*_ which is simulated.

## 3. Some Concepts Relating to AP Clustering Algorithm

### 3.1. Some Terminologies on AP Clustering

In the partitioning clustering algorithm, most techniques identify the candidate exemplars in advance by the users (e.g.,* k*-centers and* k*-means clustering). However, the affinity propagation algorithm, proposed by Frey and Dueck, simultaneously considers all data points as candidate exemplars. In the AP clustering algorithm there are two important concepts: the responsibility (*R*(*i*, *k*)) and availability (*A*(*i*, *k*)) which represent two messages indicating how well-suited a data point is to be a potential exemplar. The sum of the values of *R*(*i*, *k*) and *A*(*i*, *k*) is the evaluation basis whether the corresponding data point can be a candidate exemplar or not. Once a data point is chosen to be a candidate exemplar, those other data points with more nearer distance will be assigned to this cluster. The detailed meaning of the terminologies is as follows.

*Exemplar.* The center of a cluster is an actual data point.

*Similarity*. The similarity value between two data points *x*_*i*_ and *x*_*j*_ (*i* ≠ *j*) is usually assigned the negative Euclidean distance, such as *S*(*i*, *j*) = −‖*x*_*i*_ − *x*_*j*_‖^2^.

*Preference*. This parameter is set to indicate the preference that the data point *i* can be chosen as an exemplar, which usually is set by median(s) of all distances. In other words, preference is the willingness of the user to select the initial “self-similarity” values.

*Responsibility. R*(*i*, *k*) is an accumulated value which reflects how well the point *k* is suited to be the candidate exemplar of data point* i* and then sends from the latter to the former; that is, compared to other potential exemplars, the point *k* is the best exemplar. 

*Availability*. *A*(*i*, *k*) is opposed to *R*(*i*, *k*) and reflects how well-suited it is for the point *i* to choose point *k* as its exemplar. Based on the candidate exemplar point* k, *the accumulated message sent to the data point *i* tells it that point *k* is more qualified as an exemplar than others.

**Figure 6:**
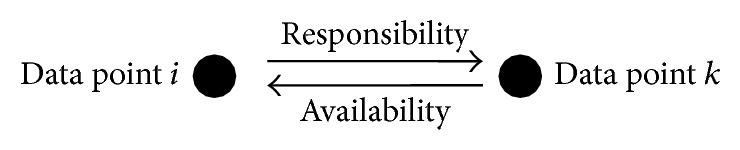
Message passing between data points.

The relationship between *R*(*i*, *k*) and *A*(*i*, *k*) is illustrated in Figure 6.

### 3.2. The Algorithm of AP Clustering

There are four main steps in AP.

**Step 1 (initializing the parameters).**

Consider

(4) $$\begin{array}{c} R\left(i,k\right)=0,\;A\left(i,j\right)=0,\;\forall i,k. \end{array}$$

**Step 2 (updating responsibility).**

Consider

(5) Ri,k=Si,k−max⁡Ai,j+Si,j,    j∈1,2,…,N;j≠k.

**Step 3 (updating availability).**

Consider

(6) Ai,k=min⁡ 0,Rk,k+∑jmax⁡0,Rj,k,       j∈1,2,…,N;j≠i, j≠k,Ak,k=Pk−max⁡Ak,j+Sk,j,       j∈1,2,…,N;j≠k.

In order to speed up convergence rate, the damping coefficient is added to iterative process:

(7) $$\begin{array}{c} R_{i+1}\left(i,k\right)=\text{lam}*R_{i}\left(i,k\right)+\left(1-\text{lam}\right)*R_{i+1}^{\text{old}}\left(i,k\right),\\ A_{i+1}\left(i,k\right)=\text{lam}*A_{i}\left(i,k\right)+\left(1-\text{lam}\right)*A_{i+1}^{\text{old}}\left(i,k\right). \end{array}$$

**Step 4 (assigning to corresponding cluster).**

Consider

(8) $$\begin{array}{c} c_{i}^{*}⟵\underset{k}{\arg\;\max}\left\{R\left(i,k\right)+A\left(i,j\right)\right\}. \end{array}$$

**Figure 7:**
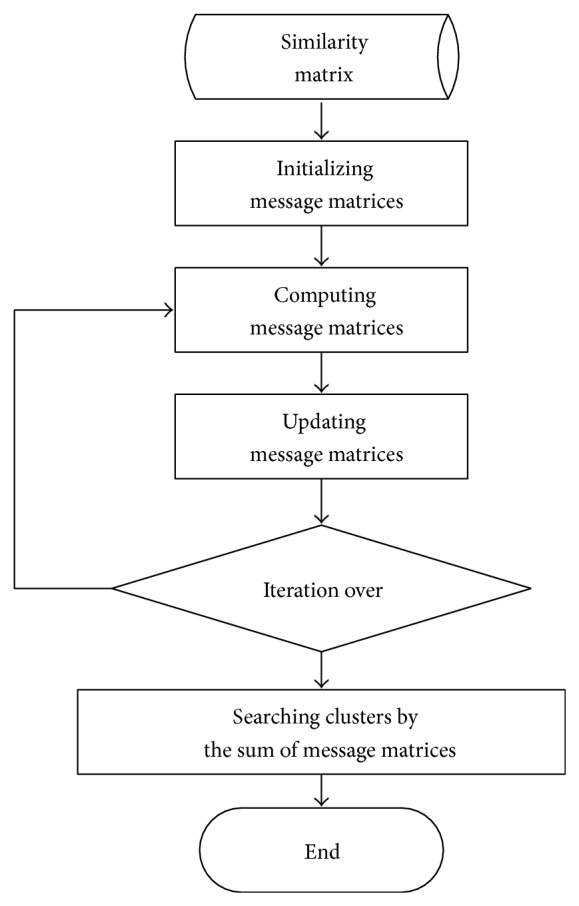
The flow diagram of extended AP algorithm.

The program flow diagram is shown in Figure 7.

## 4. The Extended AP Clustering Algorithm and Experiments

### 4.1. The Extended AP Clustering Algorithm

Based on the method of splitting the nearest distance frequency distribution by detecting the knee in the curve, we propose a semisupervised AP clustering algorithm for recognizing the clusters with different intensity in a data set as follows.

1. Calculate every data point's nearest distance (*D*_*m*_).
2. Draw the frequency distribution (FD) curve of the nearest distance matrix (*D*).
3. Analyze the FD curve and identify the partition values for obtaining the number of the density types of the whole data set.
4. Run the AP clustering algorithm within each subdataset with different data density types and extract the final clusters.

### 4.2. Experiments and Analysis

#### 4.2.1. Experiment 1

**Figure 8:**
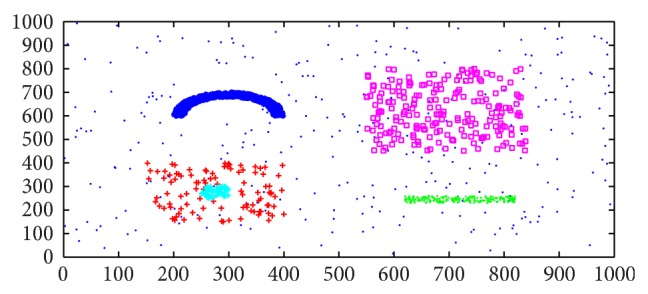
The simulated data set used in Experiment 1.

In Experiment 1, we use a simulated data set with three Poisson distributions. There are 1524 data points distributed at five intensities in a 1000 × 1000 rectangle, as shown in Figure 8. The most intensive data type includes 3 clusters (i.e., the blue, green, and Cambridge blue cluster), the medium density data type includes two clusters (i.e., the red and purple cluster), and the low density is the noise which is dispersed in the whole rectangle. We firstly applied our semisupervised AP clustering algorithm to this dataset and compared the result with the most familiar cluster method OPTICS.

**Figure 9:**
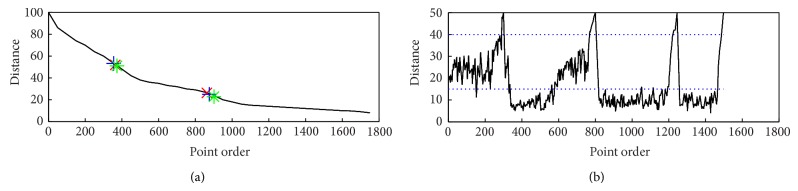
The different data density types via FD curve and reachability-plot.

According to implementing steps in Section 4.1, we calculate the nearest distance and then draw the FD curve of the distance descending as shown in Figure 9(a), which indicates 3 processes and 5 clusters existing in a simulated data set.

As a comparison, we run the OPTICS and get the reachability-plot of OPTICS (Figure 9(b)).

**Figure 10:**
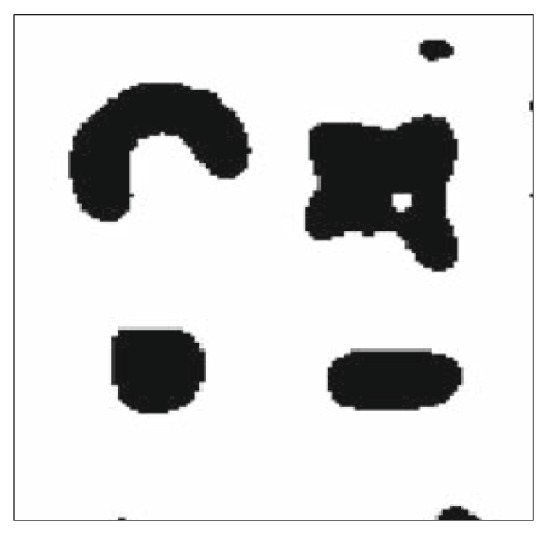
The result of Experiment 1.

After running the second step (AP clustering program) the clusters are obtained, which is clearly consistent with the expected results.  Figure 10 illustrates the clustering result.

#### 4.2.2. Experiment 2

In Experiment 2, a real seismic data set is used to evaluate our algorithm. The seismic data are selected from Earthquake Catalogue in West China (1970–1975, *M*) and Earthquake Catalogue in West China (1976–1979, *M*⩾1) [15, 16] (*M* represents the magnitude on the Richter scale). The space area covers from 100° to 107° E and from 27° to 34° N. We selected the *M*⩾2 records during 1975 and 1976.

In an ideal clustering experiment, the strong earthquakes, foreshocks, and aftershock can be clustered clearly. The former can show the locations of strong earthquakes and the latter can supply help to understand the mechanism of the earthquakes. Due to the interference of the background earthquakes it is difficult to discover the earthquake clusters. Furthermore, the earthquake records include only the coordinates of the epicenter of each earthquake. In this situation the key work is to discover the locations of the earthquakes from the background earthquakes. In this experiment we adopt the OPTICS as the comparison, which can provide a reachability-plot and can show the estimation of the thresholds.

**Figure 11:**
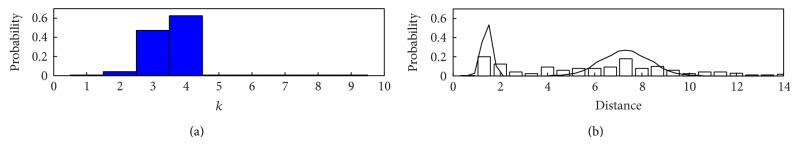
(a) The posterior probability of *k*; (b) the histogram of the nearest distance and the fitted curve.

When our AP clustering algorithm had converged, the posterior probabilities of *k* are obtained, as shown in Figure 11(a). It shows that the maximal posterior probability exists at* k* = 2. Also the histogram of the nearest distance and its fitted probability distribution function are displayed in Figure 11(b).

**Figure 12:**
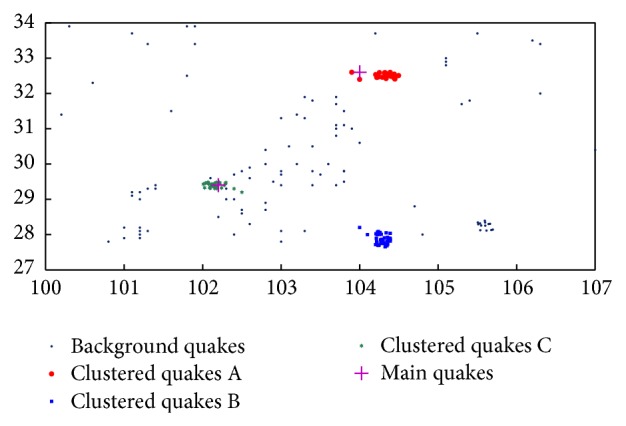
Seismic anomaly detection by our method.

**Figure 13:**
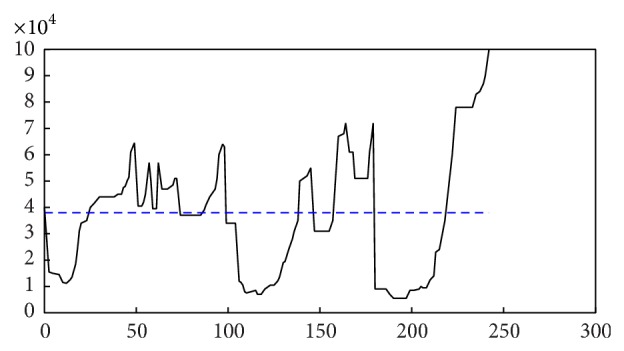
The reachability-plot of the regional seismic data.

**Figure 14:**
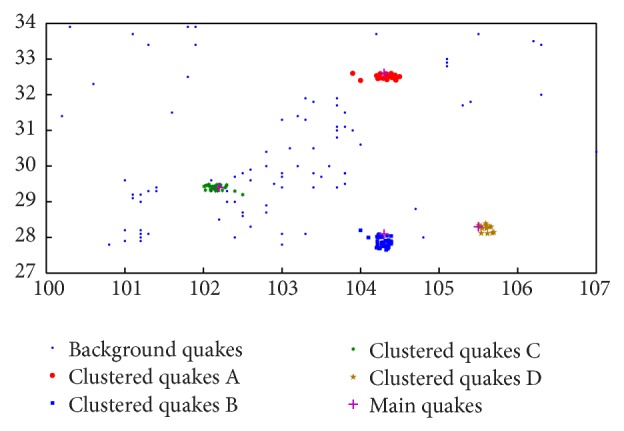
Seismic anomaly detection by OPTICS.

The experiment result is shown in Figure 12. The seismic data are grouped into three clusters: red, green, and blue. Then we apply the OPTICS algorithm to obtain a reachability-plot of the seismic data set and an estimated threshold for the classification which is shown in Figure 13. There are four grouped clusters when Eps_1_^*^ = 3.5 × 104 (m), as shown in Figure 14. This result has two different aspects from our method: firstly, there appears a brown cluster; secondly, the red, green, and blue clusters are held in common by two algorithms that contain more earthquakes in OPTICS than in our algorithm.

Further, we analyze the results of two experiments in detail. It is obvious that the clusters discovered by our algorithm are more accurate than those by OPTICS. The former clusters can indicate the location of forthcoming strong earthquakes and the following ones.

According to Figure 12, three earthquake clusters are discovered by the algorithm proposed in this paper. The red cluster indicates the foreshocks of Songpan earthquake, which occurred in Songpan County, at 32°42′N, 104°06′ E, on August 16, 1976, and its earthquake intensity is 7.2 on the Richter scale. The blue cluster means the aftershocks of Kangding-Jiulong event (*M* = 6.2), which occurred on January 15, 1975 (29°26′N, 101°48′E). The green cluster means the aftershocks of the Daguan event (*M* = 7.1), which hit at 28°06′N, 104°00′E, on May 11, 1974. Those discovered earthquakes are also detected in the work of Pei et al. with the same location and size. The difference between our method and Pei's is that the numbers of the earthquake clusters are slightly underestimated in Figure 12. The reason is that the border points are not treated in our method while in Pei et al.'s they are treated [17]. Some clusters are obtained in the border in the work from Pei et al. [18], but we can confirm that those are background earthquakes.

Finally, we compare our results with the outputs by OPTICS. After having carefully analyzed the seismic records, it can be found that the brown earthquake cluster is a false positive and the others are overestimated by the OPTICS algorithm; the blue and green clusters cover more background earthquakes.

## 5. Conclusion and Future Work

In the same data set, clusters and noise with different density types usually coexist. Current AP clustering methods cannot effectively deal with the data preprocessing for separating meaningful data groups from noise. In particular, when there are several data density types overlapping in a given area, there are more than a few existing grouping methods that can identify the numbers of the data density types in an objective and accurate way. In this paper, we propose an extended AP clustering algorithm to deal with such complex situation in which the data set has several overlapping data density types. The advantage of our algorithm is that it can determine the number of data density types and cluster the data set into groups accurately. Experiments show that our semisupervised AP clustering algorithm outperforms the traditional clustering algorithm OPTICS.

One limitation of our method is that the identification of the density types depends on the users. The following research will be focused on finding a more efficient method to identify splitting value automatically. The other aspect is to develop new AP versions to deal with the spatiotemporal data sets, as in the researches by Zhao [19, 20] and Meng et al. [21].
